# Seasonal clustering of sinopulmonary mucormycosis in patients with hematologic malignancies at a large comprehensive cancer center

**DOI:** 10.1186/s13756-017-0282-0

**Published:** 2017-12-06

**Authors:** Shobini Sivagnanam, Dhruba J. Sengupta, Daniel Hoogestraat, Rupali Jain, Zach Stednick, David N. Fredricks, Paul Hendrie, Estella Whimbey, Sara T. Podczervinski, Elizabeth M. Krantz, Jeffrey S. Duchin, Steven A. Pergam

**Affiliations:** 1 Vaccine and Infectious Disease Division, Fred Hutchinson Cancer Research Center, 1100 Fairview Ave. North, E4-100, Seattle, WA 98109 USA; 2 Department of Laboratory Medicine, University of Washington Medical Center, Seattle, WA USA; 3 Department of Pharmacy, University of Washington Medical Center, Seattle, WA USA; 4 Clincial Research Division, Fred Hutchinson Cancer Res. Ctr, Seattle, WA USA; 5 Department of Medicine, University of Washington Medical Center, Seattle, WA USA; 6 Washington State Department of Health, Shoreline, WA USA; 7 Infection Prevention, Seattle Cancer Care Alliance, Seattle, WA USA; 8 Public Health, Seattle and King County, Seattle, WA USA

**Keywords:** Mucormycosis, Fungus, Mold, Healthcare-associated infections, Immunocompromised host, Seasonal, Climate

## Abstract

**Background:**

Invasive Mucorales infections (IMI) lead to significant morbidity and mortality in immunocompromised hosts. The role of season and climatic conditions in case clustering of IMI remain poorly understood.

**Methods:**

Following detection of a cluster of sinopulmonary IMIs in patients with hematologic malignancies, we reviewed center-based medical records of all patients with IMIs and other invasive fungal infections (IFIs) between January of 2012 and August of 2015 to assess for case clustering in relation to seasonality.

**Results:**

A cluster of 7 patients were identified with sinopulmonary IMIs (*Rhizopus microsporus/azygosporus,* 6; *Rhizomucor pusillus,* 1) during a 3 month period between June and August of 2014. All patients died or were discharged to hospice. The cluster was managed with institution of standardized posaconazole prophylaxis to high-risk patients and patient use of N-95 masks when outside of protected areas on the inpatient service. Review of an earlier study period identified 11 patients with IMIs of varying species over the preceding 29 months without evidence of clustering. There were 9 total IMIs in the later study period (12 month post-initial cluster) with 5 additional cases in the summer months, again suggesting seasonal clustering. Extensive environmental sampling did not reveal a source of mold. Using local climatological data abstracted from National Centers for Environmental Information the clusters appeared to be associated with high temperatures and low precipitation.

**Conclusions:**

Sinopulmonary Mucorales clusters at our center had a seasonal variation which appeared to be related to temperature and precipitation. Given the significant mortality associated with IMIs, local climatic conditions may need to be considered when considering center specific fungal prevention and prophylaxis strategies for high-risk patients.

## Background

Fungi of the order Mucorales are ubiquitous in the environment, and known to cause life-threatening invasive infections, particularly among patients with hematologic malignancies (HM) and uncontrolled diabetes [1]. Due to the challenges of currently available diagnostics and the high levels of intrinsic resistance to many antifungal regimens, morbidity and mortality remain unacceptably high among patients who develop invasive Mucorales infections (IMI). While IMIs in immunocompromised patients usually occur as sporadic cases, there have been a number of Mucorales-related clusters and outbreaks reported in the literature [2]. The majority describe skin and soft tissue infections related to contaminated hospital supplies [3–5]; small sinopulmonary clusters, in contrast, have been less frequently reported [6–8].

In this study, first we review a cluster of Mucorales sinopulmonary infections that occurred in summer of 2014 in patients with HM, and report steps implemented to limit the development of additional cases. After an outbreak investigation, we retrospectively reviewed other IMIs in the 2 years prior to this cluster to assess overall infection rate among these high-risk patients and their association with climatic changes. Finally, we compared these data to patients in the subsequent year that included a time period during which the cluster occurred and report an additional, separate cluster of IMI cases that occurred during the summer of that year. Specifically, we hypothesized that seasonal variation of Mucorales infections, particularly during the elevated temperatures and limited precipitation of summer months, were associated with these clusters.

## Methods

### Study design

To understand the original cluster and assess our primary seasonal hypothesis, we performed a retrospective cohort study that included patients during the cluster, and the period before and after the cluster. All HM patients with IMI microbiologically-confirmed at the University of Washington Medical Center (UWMC) and Seattle Cancer Care Alliance (SCCA) between June and August 2014 defined the initial cluster. To elucidate the baseline frequency of IMIs prior to the cluster, we also reviewed all cases of IMIs in HM patients between January 2012 and May 2014. Following the cluster of IMI in 2014 and associated control interventions, in order to determine where these efforts affected risks for IMIs, we prospectively monitored for additional cases over the next 12 months, ending in August 2015. All patients with an IMI that were not related to an underlying HM, were not included in these analyses.

In a second component of the study, all HM patients with other clinically important invasive filamentous fungal infections (IFIs) (e.g. *Aspergillus* spp., *Fusarium* spp.) diagnosed between January 2013 and August 2014 were also identified; this time frame included both the initial cluster period and a referent year (calendar year 2013). Data on Posaconazole use, either for prophylaxis or treatment, was collected during the period pre and post-cluster identification, to assess center-based changes in usage.

### Setting

The SCCA/Fred Hutchinson Cancer Research Center is a National Cancer Institute-designated Cancer Center that sees approximately 75,000 patients annually, including high-risk hematopoietic cell transplant (HCT) recipients. Generally, patients commute from their permanent residence to the center or stay locally at center-based transition housing/community housing near the center. Those requiring inpatient care are admitted to the UWMC, a large 450-bed academic tertiary medical center which incorporates adult hematology and oncology units with over 100 inpatient beds.

Allogeneic HCT recipients in the initial 2 years of the study receive fluconazole as primary antifungal prophylaxis to day + 75 post transplantation. HCT recipients with high grade graft-versus-host disease (GVHD) are recommended to be given posaconazole prophylaxis unless undergoing treatment for another fungal infection [9]. Patients undergoing autologous HCT are given fluconazole until neutropenia and mucositis resolve. Prior to the cluster identification, patients with a HM at risk of prolonged neutropenia (e.g. acute myeloid leukemia [AML] and myelodysplastic syndrome [MDS]) also received fluconazole prophylaxis.

Patients admitted for treatment are managed on floors with single rooms that include high efficiency particulate air (HEPA) filtration. In the outpatient clinics, floors where HCT recipients are cared for are also HEPA filtered. Wearing a surgical mask in the outpatient department is not routine unless patients are known to have a contagious respiratory pathogen (e.g. influenza).

### Participant eligibility/case definitions

All cancer patients who were admitted to the hospital’s HCT and HM cancer units during the cohort periods of interest were included in these analyses. IFIs were defined as per the revised European Organization for Research and Treatment of Cancer/Invasive Fungal Infections Cooperative Group and the National Institute of Allergy and Infectious Diseases Mycoses Study Group (EORTC/MSG) Consensus Group definitions [10]. Histopathological, cytopathological and/or direct microscopic evidence of tissue invasive hyphal elements or culture of mold from sterile tissue were defined as proven IMIs. Cases were considered probable if they included a host factor, clinical criteria and mycological criteria. For the purposes of this study, we created an additional category, “possible with positive PCR” for IMI cases, which included those patients that fulfilled host and clinical criteria and were also positive by a laboratory-developed pan-fungal and/or Mucorales-specific PCR assays. Final analysis counted proven, probable and “possible with positive PCR” infections as cases. Patients who had only possible infection (i.e. absent mycological criteria or PCR results) were not counted as cases.

Site of infection was broadly categorized into sinus, pulmonary, cutaneous, cerebral or other. A disseminated infection referred to patients with infection involving two or more non-contiguous sites [11]. Onset of fungal infection was defined as the date at which the patient met the criteria for proven, probable or “possible with positive PCR” definition. Mortality was defined as all-cause mortality within 90 days of fungal infection. For the purposes of the analyses, summer months were considered June, July and August; fall as September, October, November; winter as December, January, February; and spring as March, April and May.

### Data sources

Cancer inpatient data, including unique patients and total inpatient-days were collected from center-based clinical databases. All patients with microbiologically-confirmed mucormycosis at the UWMC and SCCA from January 2013 – August 2015 and a second cohort of hematology/oncology patients with other clinically important IFIs from January 2013 - August 2014 were identified using laboratory records, database review and electronic medical records.

In order to address potential associations with local construction patterns, publically available construction permit data from the Seattle-King County records were assessed [12]. For the purposes of the analyses construction projects were considered active for the entire duration of the permit. Clinical cases were also compared to publically available local temperature and precipitation data gathered from Seattle Weather Service Forecast Office (WSFO) Sand Point Station (King County, WA) [13]. In order to link both sources to clinical outcomes, mean temperature and total precipitation were included in analyses. Data collection and all analyses were approved by the Fred Hutchinson Cancer Research Center (FHCRC) Institutional Review Board.

### Diagnostic methods

At our center, fungal cultures are routinely performed on all pulmonary specimens in immunocompromised hosts and on non-pulmonary specimens upon request. Diagnostic bronchoalveolar lavages (BAL) are commonly performed in patients with HM being treated with chemotherapy or those following a HCT with clinical and/or radiologic findings consistent with a pulmonary infection. Fungal cultures and Platelia™ aspergillus enzyme immunoassay (Bio-Rad Laboratories. Hercules, CA) for galactomannan (GM) are performed on all BAL samples in patients suspected to have a fungal infection; serum GM is performed on request. Any fungi growing in culture and resembling filamentous fungi are identified using conventional morphology [14] and/or DNA sequence analysis. All BAL specimens also undergo routine cytologic review for hyphal elements by center-based pathologists. In addition to standard testing from airway specimens, all lung biopsies and autopsies from patients with HM are routinely assessed for fungal infections by both histopathology and culture.

In addition, laboratory developed pan-fungal and Mucorales-specific PCRs are performed on pulmonary and non-pulmonary specimens upon request. The pan-fungal PCR is performed using published primers [15] targeting fungal ribosomal RNA genes. For the PCR specific for the Mucorales, samples are assessed using published broad range fungal primers [15] targeting the ITS2 region of the ribosomal RNA genes followed by a nested PCR with a mixture of five laboratory developed Mucorales specific primers, designed to identify *Rhizopus oryzae*, *Rhizopus microsporus* or *azygosporus*, *Mucor spp*., *Rhizomucor spp*. and *Lichtheimia corymbifera*. The analytical sensitivity of this test is 1 genome per PCR reaction. PCRs are assessed on BAL fluid, biopsy, and autopsy samples by request.

### Statistical analysis

The incidence rates of IMI were estimated by dividing the number of incident IMI cases developed in cohort subjects by the inpatient-time at-risk contributed by the overall cohort; exact 95% confidence intervals (CI) were estimated based on a Poisson distribution. Time at-risk was calculated using time during inpatient admission where patients contributed days at risk from date of admission to the date of discharge or death. Associations of season, mean monthly temperature, and total monthly precipitation with incidence rates were assessed using a Poisson regression model, with data aggregated in 1-month intervals. The number of IMI events was the dependent variable and the logarithm of the number of patient-days was included as an offset variable. Season, temperature, and precipitation were the independent variables, each included in separate univariable models. Because observed incidence rates did not increase linearly with increasing temperatures but instead showed a sharp increase at approximately 20 degrees C, we chose to model temperature as a binary variable indicating temperatures above 20 degrees C versus 20 degrees C or lower. Precipitation was modeled as a continuous variable. Model estimates were presented as incidence rate ratios (IRR) with 95% CIs. Posaconazole use was compared between early and later time periods using a chi squared test.

## Results

**Fig. 1:**
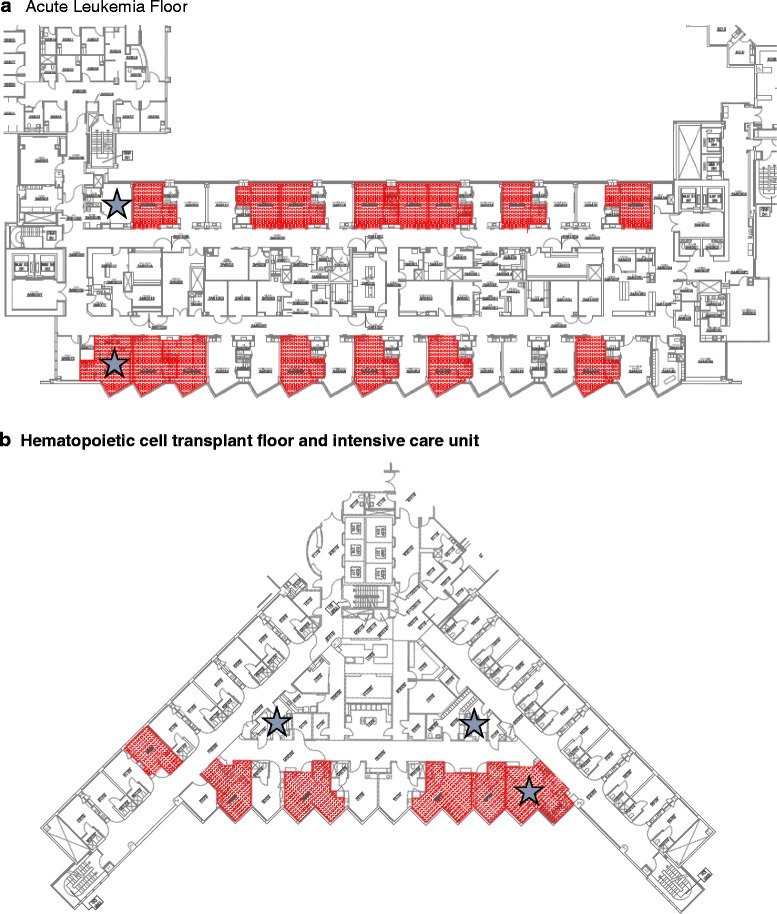
*Red areas* indicate the locations of patients involved in the first Mucorales cluster (*n* = 7). Floor plans indicate locations of individual patients over the study period of June–August 2014 in *red*. **a**. The leukemia unit. Since patients were admitted multiple times over the period of interest into multiple rooms, rooms noted in *red* are greater than the number of patients in cluster. No specific rooms were identified to be associated with cases. *Stars* indicate rooms that were designated as negative pressure rooms. Only one patient was placed into a negative pressure room during the period of interest. **b**. The hematopoietic cell transplant unit. Areas of involvement (*red*) include intensive care unit rooms, and the *blue star* indicates the negative pressure rooms. Patients in these rooms were moved to these areas after symptom onset. Only one other room was linked to this episode and included the autologous transplant recipient treated during the study period on this floor; this patient had also spent time on the leukemia unit prior to admission on this floor

Between June and August 2014, there were 907 patients admitted and 7 cases of IMIs identified, as summarized in Table 1. The median age of patients was 56 (interquartile range [IQR] 31–67) years; 4 (57%) were men. Six patients had infection reported to be *Rhizopus microsporus* or *azygosporus*, and one patient had *Rhizomucor pusillus* infection. Three had proven disease, while the others had possible disease with a positive PCR. Four patients had isolated pulmonary mucormycosis, one had sinopulmonary disease and two had disseminated disease. All patients had HM including: relapsed multiple myeloma (*n* = 1), diffuse large B-cell lymphoma (1), acute lymphoblastic leukemia (1) and refractory AML (4). Only the patient with multiple myeloma had a history of autologous HCT; no other HCT recipients were identified within this cluster of IMIs. Five patients died and two were discharged to hospice and died at a later date (Table 1). During this period of time no other patients within the larger hospital system developed IMIs. The majority of patients diagnosed with these infections were in the HM unit, likely reflecting the most at-risk population. No specific rooms, including those with negative pressure, could specifically be linked to cases (Fig. 1).

**Table 1:** Demographics, clinical features and mycological characteristics of the initial cluster of invasive Mucorale infections and the cluster the following year

Age/Sex	RF	Symptoms	Imaging	Mucorale	Mycological diagnosis	EORTC/MSG criteria	Site of infection	Prophy/Treatment at Dx	Treatment	Outcome
39/F	AML	Fever, Cough CP	Nodule, surrounding GGO	*Rhizopus microsporus* or *azygosporus*	BAL Mucorales PCR+	Poss w/PCR+	Pulmonary	Vori, Caspo (8 months)	LAMB, then posa	Death
71/M	MM, auto HCT	Fever, Skin lesions	Nodule	*Rhizopus microsporus* or *azygosporus*	Skin swab culture +; BAL pan-fungal & Mucorales PCRs+	Poss w/PCR+	Disseminated: pulmonary, cutaneous	–	LAMB and posa	Death
31/F	DLBCL	Fever Resp. failure Hypotension	Dense consolidations, pleural effusions	*Rhizopus microsporus* or *azygosporus*	BAL pan-fungal & Mucorales PCRs+; Autopsy histopath +	Proven	Pulmonary	Vori, Mica (1 month)	Nil – diagnosed after death	Death
52/M	ALL	Fever, Resp. failure	Nodule	*Rhizopus microsporus* or *azygosporus*	BAL Mucorales PCR +; Autopsy histopath & culture +	Proven	Pulmonary	Flu	Nil – diagnosed after death	Death
56/M	AML	Fever	Nodule, surrounding GGO	*Rhizopus microsporus* or *azygosporus*	BAL Mucorales PCR+	Poss w/PCR+	Pulmonary	Vori (5 months)	LAMB, then posa	Hospice
63/F	AML	Fever, Palatal lesions	Nodules w/cavitation; Diffuse sinus mucosal thickening	*Rhizopus microsporus* or *azygosporus; Mucor circinelloides*	Palate biopsy culture & histopath +; BAL culture, Mucorales & pan-fungal PCRs+	Proven	Sinopulmonary	Flu	LAMB	Hospice
73/M	AML	Chills, Resp. failure	Pulmonary nodules/consolidation; Masses w/in ventricles (Brain)	Rhizomucor pusillus	BAL Mucorales PCR+	Poss w/PCR+	Disseminated: pulmonary, CNS	Flu	LAMB	Death
50/F	ALL, HCT	Forearm skin lesion	Normal	*Rhizopus oryzae* complex	Skin biopsy culture and Mucorales PCR +	Proven	Cutaneous	Flu	Surgery, LAMB + posa, then posa	Cure
70/F	AML	Neutropenic fever, cough	Pulmonary nodules/consolidations	*Rhizopus microsporus or azygosporus*	BAL panfungal PCR+	Poss w/PCR+	Pulmonary	Posa prophylaxis ceased due to transaminitis		Death
71/M	MDS, HCT	Pulmonary nodules	Pulmonary nodules/consolidation	*Cunninghamella bertholletiae*	Autopsy histopath and Mucorales PCR+	Proven	Pulmonary	Mica	Nil	Death
45/F	AML	Neutropenic fever	Pulmonary nodules/consolidation	*Rhizomucor pusillus*	Lung tissue histopath+	Proven	Pulmonary	Posa (subtherapeutic level)	Surgery + LAMB/terbinafine/isuvaconazole	Death
59/F	MDS, HCT	Neutropenic fever, chest pain	Pulmonary nodules/kidney lesions	*Rhizomucor meihei*	Autopsy histopath, panfungal and Mucorales PCR+	Proven	Disseminated – pulmonary, renal	Vori (transaminitis), Mica	Surgery	Death

White = initial cluster in 2014; Grey = cluster in 2015, *RF* Risk factors, *EORTC/MSG* European Organization for Research and Treatment of Cancer/Invasive Fungal Infections Cooperative Group and the National Institute of Allergy and Infectious Diseases Mycoses Study Group, *Dx* Diagnosis, Resp. failure – respiratory failure, *AML* Acute Myeloid Leukemia, *CP* Chest pain, *GGO* Ground glass opacity, *CT* Chest tomography, *BAL* Bronchoalveolar lavage, *Vori* Voriconazole, *Caspo* Caspofungin, *LAMB* Liposomal amphotericin, *MM* Multiple Myeloma, *HCT* Hematopoietic cell transplantation, + positive, *PCR* Polymerase chain reaction, *DLBCL* Diffuse large B cell lymphoma, *Mica* Micafungin, *ALL* Acute lymphoblastic leukemia

### Initial management

Upon identification of the potential cluster, a comprehensive environmental assessment was undertaken to identify possible sources. Air samples were collected from multiple sites, with sites chosen based on proximity to patient rooms and entry and exit points into the inpatient and outpatient areas (e.g. elevator lobby, stairwell and hallway). Infection control teams met with the engineering departments to assess the airflow systems, which included a review of filter replacement dates. Maintenance and construction records were retrieved to determine dust risk in the 3 months prior to the detection of the first IMI case. Infection prevention teams also reviewed the historical environmental air sampling results from 2005 to 2014 (done quarterly); no evidence of major fungal pathogens had been identified during this time period. Additionally, infection control and engineering staff conducted walkthroughs of both the inpatient and outpatient facilities. A potential source for the outbreak was not identified following extensive sampling within the two hospital systems and all environmental samples remained culture negative for Mucorales*.*

**Fig. 2:**
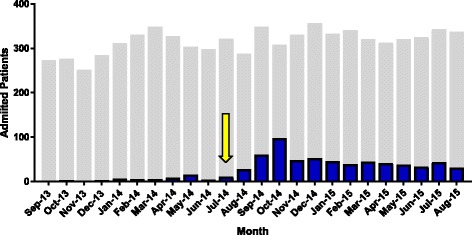
Posaconazole use (prophylaxis and treatment) prior to and after the cluster among inpatient hematology/oncology patients. The *grey bars* indicate the total number of unique inpatients during each month admitted to inpatient hematology/oncology units. *Blue bars* indicate the number of patients on posaconazole during these periods. The *yellow arrow* indicates the starting point for post-cluster interventions. Comparing Sept 2013 through August 2014 and Sept 2014 through August 2015, use of posaconazole significantly increased (90/3614 [2.5%] vs. 575/3973 [14.5%], *p* < 0.001)

In an effort to address the cluster, high-risk patients with AML and MDS undergoing chemotherapy [16] as well as any other HM with prolonged neutropenia (> 2 weeks) were recommended to receive posaconazole primary prophylaxis; posaconazole tablets were preferentially given to all patients. Figure 2 outlines the changes in posaconazole use in response to the cluster intervention, and demonstrates an increase in overall use among hematology/oncology inpatients following the identification of the cluster. In addition, a masking policy was instituted for these at-risk inpatients, where patients donned N-95 [non-fit tested] respirators when leaving the protected oncology unit while inpatient; in outpatients departments patients remained without masks as per prior center policies.

### Results from retrospective review

**Fig. 3:**
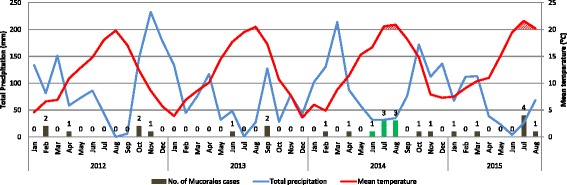
Correlation between the rates of invasive Mucorales infections and local temperature and precipitation patterns* during the initial cluster period (June – August, 2014) and the periods before and after the cluster. *Mean monthly temperature and total monthly precipitation were used for these analyses. Incidence of Mucorales infections was significantly higher during months with mean temperature above 20 degrees C (IRR, 4.64; 95% CI 2.15–10.00; *p* < 0.001) and not significantly associated with monthly total precipitation (*p* = 0.86). *Green bars* indicate the initial cluster of cases. Local temperature and precipitation data were gathered from Seattle Sand Point Weather Service Forecast Office station using the following website: http://www.ncdc.noaa.gov/cdo-web/

To compare this cluster with prior cases of IMI, we assessed a cohort of patients admitted between January 2012 and May 2014. A total of 7402 hematology/oncology patients were admitted over this period, of whom 11 patients had IMIs classified as proven, probable or possible with a positive PCR within the UWMC system. These patients were primarily proven cases (*n* = 11; proven, 8; possible with positive PCR, 3) and had a HM (AML, 5; non-Hodgkin’s lymphoma, 2; chronic lymphocytic leukemia, 1; acute lymphoblastic leukemia (ALL), 2; sickle cell disease 1); 4 were HCT recipients. *Rhizopus* spp. were most frequent (*n* = 8; *Rhizopus microsporus* or *azygosporus*, *n* = 4; *Rhizopus oryzae* complex, *n* = 2), followed by *Lichtheimia corymbifera* (*n* = 2) and *Mucor* spp. (*n* = 1). Sites of infection varied and included pulmonary (*n* = 4), sinusitis (*n* = 2), sinopulmonary (*n* = 1), gastrointestinal (*n* = 1) and disseminated (*n* = 3) infections. Only one case occurred in summer, and almost half (45%) occurred in fall (Fig. 3).

### Results from post-study period (Sep 2014 – Aug 2015)

During the immediate post-cluster period between September 2014 and August 2015, a total of 3973 hematology/oncology patients were admitted. Despite increased application of posaconazole (Fig. 2) in the year following the initial cluster there were 9 additional patients with IMIs (Fig. 3); 8 had proven disease and 1 had probable disease. This included patients with a history of AML (*n* = 3), ALL (2), MDS (2), myelofibrosis (1) and germ cell tumor (1); five of these were HCT recipients. Unlike the initial cluster, a broad range of Mucorales were identified and included: *Rhizopus microsporus* or *azygosporus* (*n* = 2), *Rhizomucor pusillus* (*n* = 2), *Mucor circinelloides* (*n* = 1), *Rhizopus* spp. unspecified (*n* = 1), *Rhizopus oryzae* complex (*n* = 1), *Cunninghamella bertholletiae* (*n* = 1) and *Rhizomucor meihei* (*n* = 1). Sites of infection included pulmonary (*n* = 6), cutaneous (*n* = 2) and disseminated (*n* = 1) IMIs. Five (56%) cases occurred in summer (Table 1, Fig. 3).Two (22%) patients were on posaconazole prophylaxis at the time of IMI. Five died and one was transferred to hospice and later died. Two patients underwent surgical resection in addition to antifungal therapy and were alive at last assessment. One additional patient was managed with pneumonectomy and triple anti-fungal therapy (amphotericin, isuvaconazole, and terbinafine) and died from relapsed disease 6 months after diagnosis.

### Other invasive fungal infections at baseline, during and after the initial cluster period

In the cohort of patients from 2013 and 2014, there were 51 HM patients (HCT, 26/51, 51%) with proven (*n* = 2) or probable (*n* = 49) aspergillosis as defined by the EORTC/MSG criteria in year 2013 and 44 cases (HCT, 12/44, 27%; proven, 2 and probable, 42) in year 2014. The highest number of cases of aspergillosis (*n* = 25) occurred between June and August 2014 when the Mucorales cluster was also documented (Fig. 3); no clear seasonal pattern was noted during the other years. In the second cohort from September 2014 to August 2015, 42 (43% in HCT recipients) cases of invasive aspergillosis were diagnosed, despite the increased use of posaconazole (Fig. 2). Five additional IFI’s occurred during the latter time period (4 *Fusarium* spp. and 1 *Scedosporium prolificans*), including one that was on posaconazole prophylaxis. There were no cases of IFIs with these species noted in our study period prior to the summer 2014.

### Seasonal and construction related assessment

A review of the local weather patterns during the cluster period revealed unusually high local temperatures and low precipitation during periods when IMI were likely to be identified (Fig. 3). When analyzed over the study cohort periods, the incidence of IMI was associated with high mean monthly temperatures (IRR, 4.64 for > 20 degrees C vs. ≤ 20 degrees C; 95% CI 2.15–10.00; *p* < 0.001), but not with mean precipitation (IRR, 1.0 per 50 mm increase; 95% CI 0.69–1.36; *p* = 0.86; Fig. 3). The incidence rates per season when including the entire study period, were 5.67 cases per 10,000 inpatient days (95% CI 3.02- 9.70) in summer, 4.10 (95% CI 1.65- 8.45) in fall, 1.94 (95% CI 0.53- 4.98) in winter, and 1.30 (95% CI 0.27- 3.81) in spring. Compared to spring, summer had significantly higher incidence rates of IMI (IRR, 4.35; 95% CI 1.24–15.27; *p* = 0.02), fall had a trend of higher rates (IRR, 3.15; 95% CI 0.81–12.16; *p* = 0.10), and winter did not have significantly different rates (IRR, 1.49; 95% CI 0.33–6.66; *p* = 0.60).

**Fig. 4:**
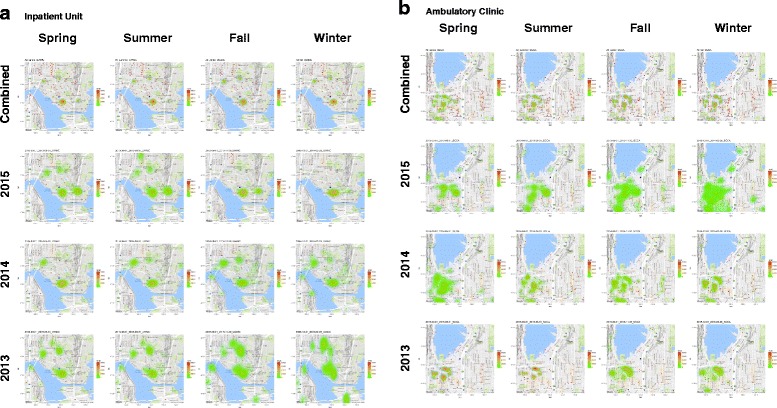
**a**/**b**: Heat map representing the locations of construction and demolition permits issued by the City of Seattle in calendar years 2014 and 2015. All data presented within 1 km of inpatient cancer units (University of Washington Medical Center [**a**] and the ambulatory clinic (Seattle Cancer Care Alliance [**b**]). *Blue central dot* indicates location of the facility

Although there were no construction activities that directly involved either the clinic or the inpatient units involved with these clusters to suggest a link, using publically available construction permits we also assessed construction sites in the areas surrounding the inpatient unit and outpatient ambulatory clinics. Within a 1 km distance from both outpatient clinic and inpatient units were areas of increased density of construction (Fig. 4 A/B). However, as these areas of construction occurred throughout the study period, and regardless of year and season, correlations between community construction sites and IMI events were not possible.

## Discussion

IMIs are rare infections that occur at an increased frequency in immunocompromised patients, and are associated with significant mortality in these high-risk populations. We described two separate clusters of IMIs in patients with HM undergoing treatment at our center during the summer of two consecutive years, involving a total of 12 patients. 83% (10/12) died or were transferred to hospice. No clear source was identified, but warm and dry summers may have contributed to these clusters. Infection control interventions that were instituted following the initial cluster did not prevent the seasonal rise in mucormycosis cases during the following summer.

Associations between weather patterns and IFIs, including those linked to mucormycosis, have been hypothesized in the literature [17–20]. Seasonal changes affect the prevalence of fungal spores in the environment [21–24], and the outdoor fungal spore counts correlate with the occurrence of IFIs [25]. The sporangiospores from Mucorales are small and can readily aerosolize and disperse throughout the environment, given favorable climatic conditions [26], thereby predisposing susceptible hosts to develop invasive disease. The precise seasonal pattern that is associated with IFIs may vary between different countries and regions; in Seattle, WA (USA) high fungal spore counts have been associated with low precipitation and high temperature [22]. We hypothesize that the two clusters of IMIs seen at our center may have been secondary to hot and dry summer conditions, conducive for both the aerosolization, spread and even virulence [27] of these invasive fungi in outpatient community environments, as was observed over this two year period.

Multiple air and surface samples within the hospital and outpatient clinic did not identify any Mucorales; air sampling outside the outpatient clinic was also culture negative. Negative results however, do not rule out the potential for a common source within either the outpatient or inpatient facility. Prior studies have shown that airborne fungal levels can range anywhere from 1 to 659 CFU/m^3^ during outbreaks [28], with testing sites and activities around these sites affecting the fungal spore counts [24]. Even with appropriate sampling, the yield from environmental sampling may be poor as the optimal incubation temperature necessary to isolate specific Mucorales in laboratory can vary widely [28]. Although air sampling did not detect Mucorales in our study, given only a small number of patients had skin infections, it was felt that exposure was still more likely to be airborne community exposures rather than related to contact with infected hospital supplies [8]. Laundry services, which have been more recently linked to pulmonary outbreaks of these infections [8], were unfortunately not assessed during the investigation. Although preventive measures such as unit-based HEPA filters were in place within high-risk wards, multiple opportunities for exposures to aerosolized spores from the outside environment were also possible, during in-hospital transfers, in ambulatory settings and through outpatient community exposures [29].

Hospital construction is commonly linked to fungal outbreaks [30], but there were no construction projects at either center that were thought to be linked to these events. In contrast, the local population is growing rapidly and available construction permits suggest that there were high-levels of construction near facilities throughout the pre- and post-cluster periods (Fig. 4a/b). Seasonal construction combined with low precipitation and higher temperatures, may have provided more opportunities for patient community exposures. It is also possible that differences in construction activities (e.g. structural work versus ground breaking) may be seasonal. Regardless, the diversity of community construction projects often present in large metropolitan cancer centers make establishing links between specific sites and patient outcomes very challenging, and prevention strategies aimed at protecting patients from such exposures can be difficult.

The infection control measures that we instituted for the control of the cluster included the introduction of posaconazole prophylaxis for high-risk patients, and the institution of N-95 masks for high-risk inpatients leaving the protected hematology unit. The number of cases of IMIs during the immediate follow-up period declined; however, it is unclear if this was a direct result of these prevention efforts. Interestingly, these same infection prevention strategies did not prevent a similar increase in  IMIs in the summer of 2015. Despite a rise in overall use of posaconazole at our center in response to the initial cluster, only 2 patients were on posaconazole prophylaxis at the time of IMI diagnosis in the post-study period. Significant out-of-pocket expenses associated with posaconazole use for patients, drug interactions and intolerance were some of the factors that limited posaconazole use in some patients. Given the high mortality rates associated with IMIs, further studies are needed to determine whether short term use of posaconazole, perhaps during high-risk time periods, may be a suitable option for at-risk patients. Of note, none of the patients had a history of allogeneic HCT in the initial cluster. It is possible that more limited periods of neutropenia post-HCT typically seen in these patients, more limited use of high-dose glucocorticoids or the possible benefit of standardized posaconazole prophylaxis in those patients during high-risk periods (e.g. GVHD) may have played a role in protecting these patients.

The move towards increased use of posaconazole prophylaxis did not significantly alter the incidence of invasive aspergillosis, as has been seen by others [31]. Interestingly, the number of cases of *Fusarium* spp. infections rose from a baseline of no cases prior, to 4 cases in the post-cluster period. This included one patient who developed disseminated *Fusarium solani* infection while on posaconazole prophylaxis. Two additional patients developed IMIs while on posaconazole prophylaxis. As discussed previously, due to the inconsistent use of prophylaxis in all high-risk patients, we cannot comment on the overall beneficial effects of posaconazole prophylaxis as has been seen by other studies [9, 16, 32]. Breakthrough Mucorales infections in patients on posaconazole prophylaxis [33], and the emergence of azole-resistant aspergillus species reported by other groups [34] are concerning and suggest a need to evaluate trends in both the incidence of infection and resistance among filamentous molds in future studies. As recent reports have shown increasing rates of fungal infections in high-risk patient populations [35], prospective studies are needed to address benefits of the new oral posaconazole tablet formulations when used for prophylaxis.

There are a number of limitations to our retrospective study. Given the rarity of these infections, the total case numbers remain small, limiting our ability to make strong associations, assess unique risk factors for the development of IMIs or make more clear links to climatic conditions. We identified cases based on positive microbiology results, meaning patients with possible IMI or other IFI without positive microbiological result (e.g. patients with host factor and abnormal imaging finding without a microbiological diagnosis, etc.) who may have also had a mold infection, were not considered cases in these analyses. No samples related to the cluster were available for additional sequencing, which might have allowed a more in-depth outbreak evaluation. Importantly, as laundry services and links to sinopulmonary IMI were not well described at the time of this investigation, this possible association was not directly assessed. Finally, most of our cases would not have met EROTC/MSG criteria, as many cases in the cluster were found only through a laboratory developed and internally validated PCR. However, these data suggest variance in IMI incidence that may be related to local weather patterns [36], and suggest a need to consider changes in current climate models and the potential for increased seasonal clusters of IMIs in high risk immunocompromised patients.

## Conclusions

In summary, this study highlights possible seasonal association of IMIs in high-risk patients with HM at our center. Given the lack of standardized guidelines for investigation, mitigation and prevention of Mucorales clusters among high-risk patients, the evaluation and management of possible outbreaks can be vexing for Infection Prevention teams. Although determining no clear common source, we made precautionary changes to our standard antifungal prophylaxis and instituted universal N-95 mask use for high-risk patients leaving the hematology wards. Despite these efforts, we saw a similar seasonal increase in cases of IMIs in the following summer. As rises in currently observed and projected global temperatures, shifts in precipitation patterns and extreme climatic events [36] become more frequent throughout the world, centers with immunosuppressed patients at risk for IMIs should be wary of possible shifts in fungal risk for their patients. Understanding local epidemiologic patterns of invasive fungal infections and the role of seasonal changes may help direct empiric therapy and guide future programs aimed at preventing fungal infections.

## Abbreviations

ALL: Acute lymphoblastic leukemia
AML: Acute myeloid leukemia
BAL: Bronchoalveolar lavage
EORTC/MSG: European Organization for Research and Treatment of Cancer/Invasive Fungal Infections Cooperative Group and the National Institute of Allergy and Infectious Diseases Mycoses Study Group
GM: Galactomannan
GVHD: Graft versus Host Disease
HCT: Hematopoietic cell transplant
HEPA: High efficiency particulate air
HM: Hematologic malignancies
IFI: Invasive filamentous fungal infections
IMI: Invasive Mucorales Infections
IQR: Interquartile range
IRR: Incidence rate ratio
MDS: Myelodysplastic syndrome
PCR: Polymerase chain reaction
SCCA: Seattle Cancer Care Alliance
SOT: Solid organ transplant
UWMC: University of Washington Medical Center
